# Differences in Capacity for Wonder and Tolerance for Ambiguity by High School of Origin

**DOI:** 10.31662/jmaj.2025-0243

**Published:** 2025-09-19

**Authors:** Hirohisa Fujikawa, Takayuki Ando, Junji Haruta

**Affiliations:** 1 Department of General Medicine, Juntendo University Faculty of Medicine, Bunkyo-ku, Tokyo, Japan; 2 Center for General Medicine Education, School of Medicine, Keio University, Shinjuku-ku, Tokyo, Japan; 3 Department of Medical Education Studies, International Research Center for Medical Education, Graduate School of Medicine, The University of Tokyo, Bunkyo-ku, Tokyo, Japan; 4 Medical Education Center, School of Medicine, Keio University, Shinjuku-ku, Tokyo, Japan

**Keywords:** capacity for wonder, tolerance for ambiguity, high school of origin, admission, medical school

## Introduction

In Japan, students are eligible to enter medical school upon graduation from senior high school or an equivalent educational program ^(1)^. Consequently, the majority of medical students enter medical school at ages 18-20 years ^(1), (2)^. Many of the entrance examinations for medical school are highly competitive ^(1)^, with pass or fail criteria that appear to be heavily dependent on academic ability or cognitive performance ^(3), (4)^. Conversely, several universities in Japan have adopted a system that allows students to go on to study at the university’s medical school after graduating from the affiliated high school. It is generally acknowledged that the system alleviates the burden on students to develop advanced cognitive abilities, such as by exempting them from written examinations.

X University (name withheld for anonymity) School of Medicine enrolls 110 medical students each year, approximately 60% of whom enter from non-affiliated high school and 40% from affiliated high school. From our daily interactions with medical students at X University, from affiliated and non-affiliated high schools, the following question has arisen: Are there any differences in attributes between medical students who graduated from the affiliated high schools and those who entered through the entrance exam from outside the affiliated high schools? This question was based on the assumption that, given the intense competition and demanding cognitive abilities necessary for gaining admission to medical school, as previously outlined, the content of education and school life would depend on whether the high school is affiliated with the medical school. This study focused on two key non-cognitive attributes: capacity for wonder (CfW) and tolerance for ambiguity (TFA). CfW, defined as a personal disposition to experience wonder consistently on fitting occasions, is likely to facilitate lifelong learning, a characteristic highly valued among health care professionals ^(5)^. TFA is defined as “the tendency to perceive situations as desirable” ^(6)^ and is associated with a range of positive outcomes, including reduced risk of burnout ^(7)^, increased empathy ^(8)^, and improved quality of patient care ^(9)^. Although they appear to be overlooked in entrance examinations, both non-cognitive attributes have recently gained substantial attention within the field of medical education. We believe that in-depth understanding of CfW and TFA is imperative for the advancement of the field, given their potential contributions to lifelong learning, better mental health, and quality patient care.

Thus, we aimed to examine the differences in CfW and TFA by high school of origin.

## Methods

### Study design and participants

This study was conducted at X University School of Medicine in May 2024 in Japan as part of research project on medical professionalism. We asked all medical students of the university to participate in the study via an electronic bulletin board system and complete an online questionnaire on SurveyMonkey (www.surveymonkey.com). Only those who showed a willingness to participate were included.

### Outcome variables: CfW and TFA

The outcome variables included CfW and TFA. We evaluated CfW using the Japanese version of the CfW scale (J-CfWS) and TFA using the Japanese version of the TFA scale (J-TFAS) ^(10), (11), (12), (13)^. Both scales have good reliability and validity ^(10), (11), (12), (13)^. The J-CfWS comprises nine items ^(11)^, each of which is rated on a 6-point Likert scale, ranging from 1 = not at all likely to 6 = extremely likely ^(11)^. The CfWS score is calculated by simply summing up the responses to the nine items, giving a possible range of 9 to 54, with higher scores corresponding to greater CfW ^(11)^. The J-TFAS has seven items, each of which is answered on a 6-point Likert scale, ranging from 1 = strongly agree to 6 = strongly disagree ^(10)^. The TFAS score is the sum of responses to the seven items, giving a possible range of 7 to 42, with higher scores indicating greater TFA ^(10)^.

### Explanatory variable: high school of origin

A dichotomous variable was created and treated as an explanatory variable: 1 = affiliated high school and 0 = non-affiliated high school.

### Statistical analysis

We performed descriptive statistics. Unadjusted associations between high school of origin and participant characteristics were analyzed by the χ^2^ test for categorical variables or Student’s t test for continuous variables.

We then performed multivariable linear regression analysis, controlling for gender and year group. A two-sided p-value less than 0.05 was considered statistically significant. We chose complete-case analysis. SPSS version 29.0.2.0 (IBM Corp) was used for statistical analysis.

### Ethical statement

The study was approved by the ethics committee of Keio University School of Medicine (20231223). Participants provided written informed consent by ticking the consent box in the online survey.

## Results

Of the 669 eligible participants, 181 agreed to participate. Four had missing data, and the remaining 177 (26.5%) were included in the final analysis. Table 1 depicts the profile of the participants. The majority were male (115, 65.0%), were in a non-clinical-year group (159, 89.8%), and entered medical school from non-affiliated high schools (102, 57.6%).

**Table 1.** Characteristics of the Participants.

Characteristics	Total (N = 177)	Non-affiliated high school (N = 102)	Affiliated high school (N = 75)	p-Value^a^
Gender, n (%)				0.09
Female	62 (35.0)	41 (40.2)	21 (28.0)	
Male	115 (65.0)	61 (59.8)	54 (72.0)	
Year group, n (%)				0.41
Non-clinical year	159 (89.8)	90 (88.2)	69 (92.0)	
Clinical year	18 (10.2)	12 (11.8)	6 (8.0)	
J-CfWS^b^, mean (SD)	35.0 (8.2)	35.2 (7.5)	34.8 (9.2)	0.71
J-TFAS^c^, mean (SD)	23.0 (6.3)	23.5 (6.3)	22.4 (6.3)	0.29

CfW: capacity for wonder; J-CfWS: Japanese version of the capacity for wonder scale; J-TFAS: Japanese version of the tolerance for ambiguity scale; SD: standard deviation; TFA: tolerance for ambiguity. ^a^By χ^2^ test for categorical variables or Student’s t test for continuous variables. ^b^Ranging from 9 to 54, with higher scores corresponding to greater CfW. ^c^Ranging from 7 to 42, with higher scores corresponding to greater TFA.

Table 2 shows the results of the multivariable linear regression analysis to examine differences in J-CfWS and J-TFAS score by high school of origin. After controlling for gender and year group, there were no significant score differences in CfW or TFA between students from affiliated high schools and those from non-affiliated high schools.

**Table 2.** Associations of High School of Origin with CfW and TFA.

	Unadjusted mean difference (95% CI)	p-Value	Adjusteda mean difference (95% CI)	p-Value
J-CfWS^b^				
Non-affiliated high school	Ref.	Ref.	Ref.	Ref.
Affiliated high school	-0.47 (-2.95 to 2.01)	0.71	-0.49 (-3.00 to 2.03)	0.70
J-TFAS^c^				
Non-affiliated high school	Ref.	Ref.	Ref.	Ref.
Affiliated high school	-1.02 (-2.92 to 0.88)	0.29	-1.28 (-3.19 to 0.63)	0.19

CfW; capacity for wonder; CI: confidence interval; J-CfWS: Japanese version of the capacity for wonder scale; J-TFAS: Japanese version of the tolerance for ambiguity scale; Ref.: reference category; TFA: tolerance for ambiguity. ^a^Adjusted for gender and year group. ^b^Ranging from 9 to 54, with higher scores corresponding to greater CfW. ^c^Ranging from 7 to 42, with higher scores corresponding to greater TFA.

## Discussion

This study investigated the score differences in J-CfWS and J-TFAS between students from affiliated high schools and those from non-affiliated high schools. The multivariable linear regression analysis revealed no significant score differences. Our findings will provide an insight into appropriate admissions policy for medical students.

To the best of our knowledge, only a few studies have elucidated the relationship between high school of origin (or admission pathway) and their attributes, and their findings were mixed. A recent US study showed that although students from higher-performing high schools perform better in the early years of medical school, these initial differences often diminish during medical school, with students from a variety of backgrounds performing similarly by the end of study ^(14)^. Conversely, an Iranian study revealed that high school entrants were less resilient and had lower self-awareness and self-control than graduate entrants, despite their greater cognitive abilities ^(15)^. These mixed findings highlight the importance of considering differences in educational systems and culture. In Japan, the majority of medical students are aged between 18 and 20 upon entering school ^(1), (2)^, and the entrance examinations are of a highly competitive nature ^(1)^, with cognitive ability accorded significant weight. Further research in this area, which takes into account these contextual factors, will facilitate the development of more effective systems.

The point estimates of affiliated high school were all negative, although not statistically significant. The results were noteworthy and unexpected given our assumption that the system of enrollment from the affiliated high school would reduce the students’ burden of developing advanced cognitive abilities and possibly enhance CfW/TFA. Further studies are necessary to clarify the mechanisms.

Finally, we note some study limitations. First, the response rate was relatively low. It is possible that students with lower CfW or TFA did not answer the questionnaire. Future studies should try to maximize the response rate. Second, the number of the participants in analysis was relatively low. Particularly, few clinical-year medical students completed the questionnaire. The small sample size would possibly lead to inadequate statistical power and, thus, yield non-significant results. Third, this was a single-center study. Further multicentered research to address our research question is recommended.

## Conclusions

High school of origin was not associated with CfW or TFA among medical students in Japan. Further multicentered studies are warranted to support our conclusions. The findings will inform appropriate policies for medical student admissions.

## Article Information

### Acknowledgments

The authors would like to take this opportunity to thank the study participants.

### Author Contributions

Hirohisa Fujikawa conceived the study, with input from Takayuki Ando and Junji Haruta. Hirohisa Fujikawa conducted the data analysis and drafted the manuscript. All authors discussed, proofread, and approved the final version of the manuscript.

### Conflicts of Interest

None

### Ethical Approval Statement

The study was approved by the ethics committee of Keio University School of Medicine (20231223). Participants provided written informed consent by ticking the consent box in the online survey.
